# Enhancing the Source-Side Transient Response of Logarithmic Double-Spiral Terahertz Photoconductive Antennas Using a Central-Feed-Boundary Tip Array

**DOI:** 10.3390/mi17091053

**Published:** 2026-09-03

**Authors:** Chao Xu, Shuaiqi Wang, Yu Zhang, He Dong, Jian Gao, Guofeng Wang

**Affiliations:** 1 College of Information Technology, Jilin Engineering Research Center of Optoelectronic Materials and Devices, Jilin Normal University, Siping 136000, China; 15582153348@163.com (C.X.);; 2 Jilin Provincial Key Laboratory of Wide Bandgap Semiconductor Material Growth and Device Applications, Jilin Normal University, Changchun 130103, China; 3 Changchun Observatory, National Astronomical Observatories, Chinese Academy of Sciences, Changchun 130117, China

**Keywords:** photoconductive antenna, logarithmic double-spiral, tip array, source-side response

## Abstract

The smooth central feed boundary of a logarithmic double-spiral terahertz photoconductive antenna can limit the spatial overlap between the high-field region and photogenerated carriers, thereby restricting the source-side transient response. We introduced a micrometer-scale tip array along the central feed boundary and investigated the influence of tip apex angle, height, and spacing through a transient semiconductor model in COMSOL Multiphysics. The photocurrent derivative, which is proportional to the emitted terahertz electric field under the transient-current approximation, was adopted as the source-side response metric. The tip array enhanced the response by redistributing the local bias field and bringing the carrier-collection boundary closer to the illuminated region. Within the investigated ranges, tip height produced the strongest response variation, whereas the influence of the apex angle remained limited over 10–50°, and further reducing *d* from 0.5 to 0.25 μm resulted in only a marginal response improvement. For *θ* = 30°, *h* = 1.0 μm, and *d* = 0.5 μm, the positive-peak and peak-to-peak enhancement ratios reached 100.1% and 103.9%, respectively. These results provide source-side design guidance for parameter selection while qualitatively considering geometric perturbation and fabrication practicality.

## 1. Introduction

Terahertz (THz) waves typically refer to electromagnetic waves with frequencies between microwaves and infrared, and they have significant applications in high-speed communication, non-destructive testing, biomedical imaging, and materials characterization [1,2,3,4]. However, the development of THz technology remains limited by radiation efficiency and device performance due to the lack of efficient solid-state THz sources. Photoconductive antennas (PCAs) utilize ultrashort laser pulses to excite semiconductor materials to generate transient photocurrents, which in turn generate THz radiation through the rapid movement of charge carriers under an applied bias electric field [5,6,7,8]. PCAs are currently one of the important radiation sources widely used in THz time-domain spectroscopy systems.

The THz response of a photoconductive antenna is mainly influenced by the generation, transport, and collection processes of photogenerated carriers [7,8]. Among these, the electrode structure determines the local bias electric field distribution and the location of the carrier collection boundary, playing a crucial role in the formation of transient photocurrent. Therefore, optimizing the electrode structure and local geometry to improve carrier dynamics is an important research direction for enhancing PCA performance [9,10,11].

In recent years, researchers have proposed various structural optimization methods to enhance the performance of photoconductive antennas. For example, increasing the effective contact length of the electrodes or optimizing the electrode arrangement can improve the carrier collection process; introducing plasmonic contact electrodes, metallic nanostructures, and periodic electrode structures can enhance the local optical field and improve the photogenerated carrier generation efficiency [12,13,14,15,16,17,18]. Furthermore, logarithmic spiral photoconductive antennas, due to their self-similar structure and broadband response characteristics, are widely used in THz source design [19,20,21]. However, existing research mainly focuses on electrode materials, nanoscale optical field enhancement, and overall antenna structure optimization. A systematic study on how the boundary morphology of the central feed region affects the coupling relationship between photogenerated carriers, electric field distribution, and the carrier collection process remains lacking.

In traditional logarithmic double-helix photoconductive antennas, the center-feed region typically employs a relatively smooth electrode boundary structure. Since photogenerated carriers are mainly concentrated in the laser-irradiated region, while the strong bias electric field region is primarily distributed near the electrode edges, a spatial mismatch may exist between the two, thus limiting the contribution of some photogenerated carriers to the transient current. Therefore, adjusting the morphology of the center-feed boundary can better match the local electric field distribution with the photogenerated carrier region.

To address the aforementioned issues, this paper proposes a micrometer-scale tip array structure based on center-fed boundary modulation and incorporates it into a logarithmic double-helix photoconductive antenna. Unlike methods that alter the overall antenna size or introduce additional optical enhancement structures, this paper optimizes only the local boundary of the center-fed region to investigate the impact of boundary geometry changes on the source-end transient response. A transient semiconductor model is established based on COMSOL Multiphysics to systematically analyze the influence of tip angle, height, and spacing on the photocurrent derivative response, further revealing the physical mechanisms by which the tip structure modulates the electric field distribution, carrier transport, and collection processes. The proposed local boundary optimization strategy is expected to provide a feasible design approach for PCA-based terahertz time-domain spectroscopy systems, particularly for improving the source-side transient response of broadband THz emitters.

## 2. Model and Simulation

### 2.1. Antenna Geometry and Tip Array Structure

We used the semiconductor module in COMSOL Multiphysics 6.4 to establish a transient carrier transport model for a logarithmic double-helix photoconductive antenna. This model mainly studies the source-side transient response of the photoconductive antenna.

The antenna model is built on a square GaAs substrate with a side length of 250 μm, and Au electrodes are placed on the substrate surface. The logarithmic double-helix electrode is constructed using parametric curves, where *φ* is a curve parameter. The two parametric curves constituting the first electrode are defined as follows:

(1) $$\begin{array}{lll} x_{1}=R_{1}\times e^{\left(a_{1}\times \varphi \right)}\times \cos\varphi & \;y_{1}=R_{1}\times e^{\left(a_{1}\times \varphi \right)}\times \sin\varphi & \;0\leq \varphi \leq 2\pi N\\ x_{2}=R_{1}\times e^{\left(a_{1}\times (\varphi -\pi /2)\right)}\times \cos\varphi & \;y_{2}=R_{1}\times e^{\left(a_{1}\times (\varphi -\pi /2)\right)}\times \sin\varphi & \;\pi /2\leq \varphi \leq 2\pi N \end{array}$$

where *R*_1_ = 5.0 μm is the initial radius, *N* = 1.5 is the number of turns, and *a*_1_ = 0.31 is the radial expansion coefficient. The first Au electrode is formed by connecting the two parameter curves. The second electrode is obtained by rotating the first electrode 180° around the center of the antenna, thus forming a centrally symmetric logarithmic double-helix electrode structure.

To investigate the influence of local boundary geometry on the source-side transient response, five micrometer-scale tips were introduced along the inner boundary of each electrode, while the outer spiral arms remained unchanged. The tip-array geometry was characterized by three parameters: the apex angle *θ*, tip height *h*, and tip spacing *d*.

**Figure 1:**
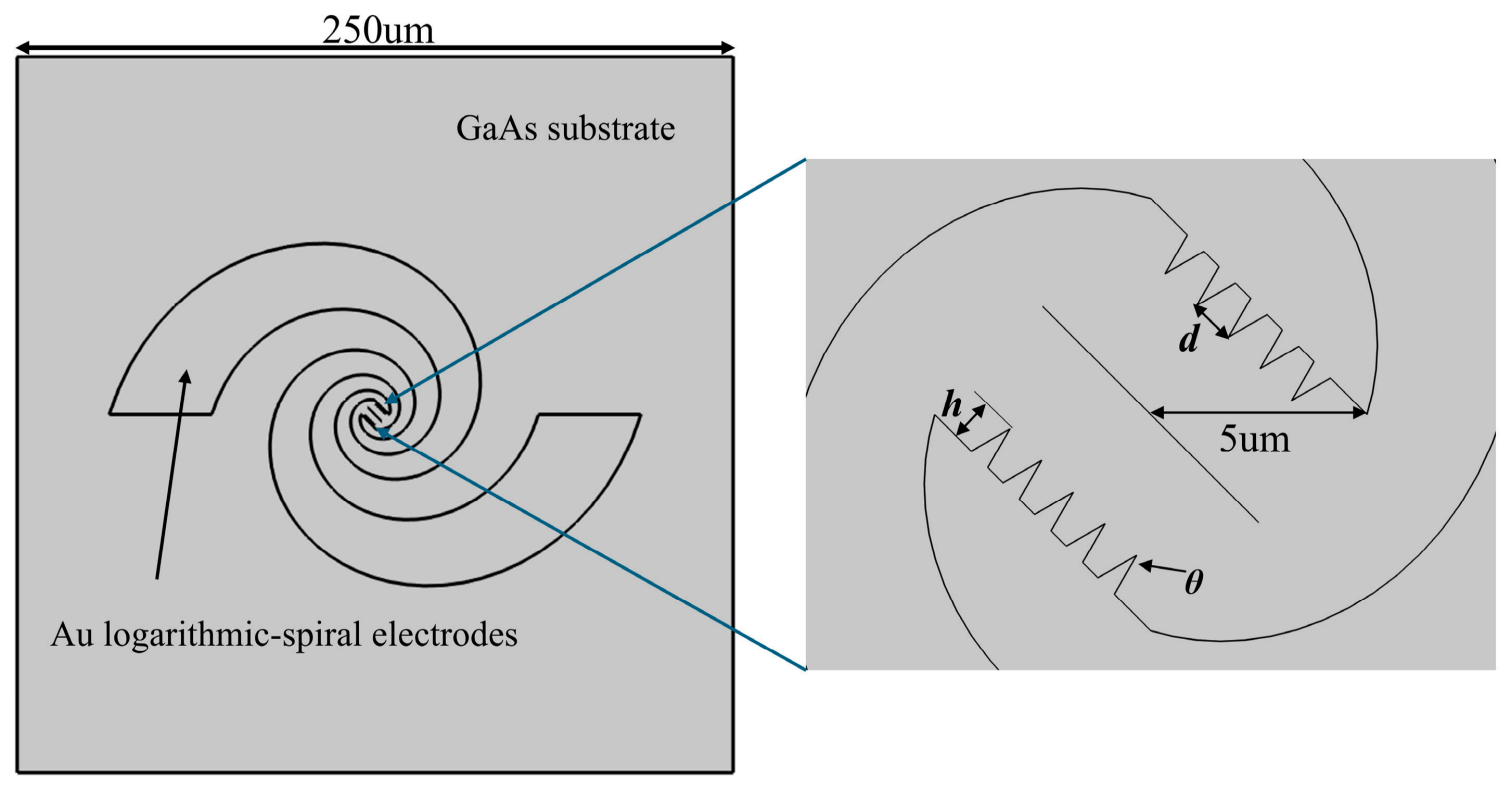
Geometric model of the photoconductive antenna.

The overall antenna geometry and the enlarged central feed region are shown in Figure 1.

### 2.2. Material Selection and Semiconductor Physics

GaAs and Au were chosen as the photoconductive substrate and metal electrode materials, respectively. GaAs was selected because of its wide application in photoconductive terahertz devices and its suitable light absorption and carrier transport characteristics under near-infrared femtosecond laser excitation. This study used a 780 nm laser for photoexcitation, as the photon energy at this wavelength is higher than the bandgap energy of GaAs, thus enabling the generation of photogenerated electron–hole pairs in the illuminated region. The higher carrier mobility of GaAs facilitates the rapid formation of transient photocurrents by photogenerated carriers under a bias electric field. Au has high electrical conductivity and good compatibility with common metal patterning processes; therefore, it was chosen as the electrode material.

The semiconductor physics model includes processes such as metal contact, optical transitions, and trap-assisted recombination. A 40 V potential difference is applied between the two electrodes to establish the initial bias electric field and drive the transport and collection of photogenerated carriers. The optical transition process describes the generation of photogenerated carriers, while the carrier recombination process is described using the Shockley–Read–Hall model. The model describes the generation, transport, and recombination of photogenerated carriers by solving the carrier continuity equation, carrier transport equation, and electrostatic equation in a coupled manner [22,23,24].

We used a Gaussian laser pulse with a wavelength of 780 nm, a peak intensity of 1 mW/μm^2^, and a spatial beam radius *w*_0_ = 2.5 μm for optical excitation. The spatial and temporal distributions of the optical pulse were defined by functions an_1_(*x*, *y*) and g_1_(*t*), respectively. The pulse time center was set to 0.5 ps, and the standard deviation was set to 100 fs.

All antenna structures use the same material parameters, bias voltage, and optical excitation conditions to ensure that the response differences between different structures mainly originate from variations in the geometric parameters of the tip array.

### 2.3. Numerical Solution and Source-Side Response Evaluation

We used the semiconductor module in COMSOL Multiphysics to solve the model. Due to the drastic changes in the electric field and carrier distribution near the center feed gap and the tip, we refined the local mesh in these areas and ensured that the calculation results were not significantly affected by the mesh size through mesh independence checks.

The calculation process is divided into two stages: steady-state solution and transient solution. First, a steady-state solution is performed with the optical transition process disabled to obtain the initial electric field and carrier states after applying a 40 V bias voltage. Then, a transient solution is performed with the photoexcitation process enabled. The simulation time range is 0–10 ps, and the time step is 0.0125 ps.

In the transient calculation process, physical quantities such as electron concentration, hole concentration, electric potential, electric field distribution, photocurrent density, and total electrode current are extracted. The total electrode current is obtained through the boundary current variable semi.I0_1.

Under the transient-current approximation, the radiated terahertz electric field is approximately proportional to the temporal derivative of the transient terminal current [25]:

(2) $$E_{THz}(t)\propto dI(t)/dt$$

Accordingly, the extracted current waveform *I*(*t*) was differentiated with respect to time to obtain *dI*(*t*)/*dt*. The maximum positive value and peak-to-peak amplitude of the resulting waveform were used to evaluate the source-side transient response. The maximum positive value represents the largest rate of current increase after optical excitation, whereas the peak-to-peak amplitude characterizes the overall variation in the derivative waveform. These two metrics were used to compare different tip-array geometries.

## 3. Results and Discussion

### 3.1. Field and Photocurrent Characteristics of the Tip-Free Reference Structure

We first simulated the tip-free logarithmic double-spiral PCA with *R*_1_ = 5.0 μm and treated it as the reference for all tip-array configurations.

**Figure 2:**
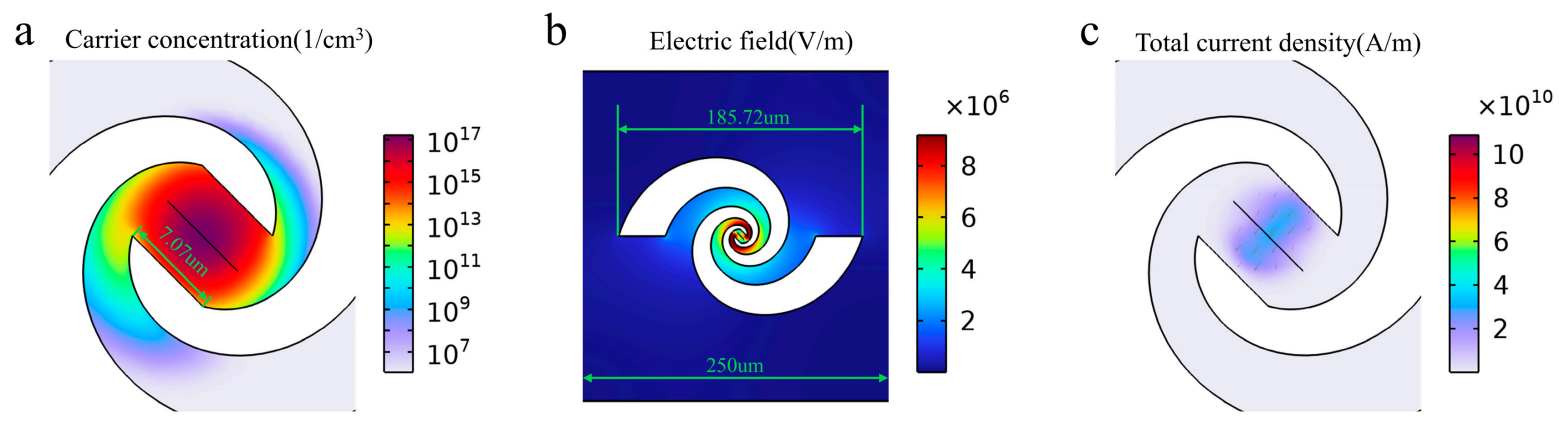
Simulation results for the tip-free logarithmic double-spiral photoconductive antenna: (**a**) electron concentration distribution; (**b**) electric field distribution; (**c**) photocurrent density distribution.

As shown in Figure 2, photogenerated carriers are mainly concentrated in the central feed region. Therefore, carrier transport and photocurrent formation are directly governed by the electric-field distribution and boundary morphology in this region.

Although the tip-free structure can generate a basic photocurrent response, its strong electric field is mainly confined to the vicinity of the smooth electrode edges. It does not sufficiently overlap with the high-carrier-concentration region formed by central illumination. Consequently, some photogenerated carriers cannot be effectively accelerated and collected by the strong electric field. These distributions suggest that the smooth central feed boundary provides limited overlap between the high-field region and the photoexcited carrier population, thereby motivating local modification of the feed boundary.

### 3.2. Influence of Tip Apex Angle on the Source-Side Transient Response

In the tip-array structure, the apex angle influences the degree of local field concentration near the tips. To isolate the influence of the apex angle, *h* = 1.0 μm and *d* = 0.5 μm are kept constant, while *θ* is set to 10°, 20°, 30°, 40°, and 50°. We then compared the corresponding source-side responses with that of the tip-free reference structure.

**Figure 3:**
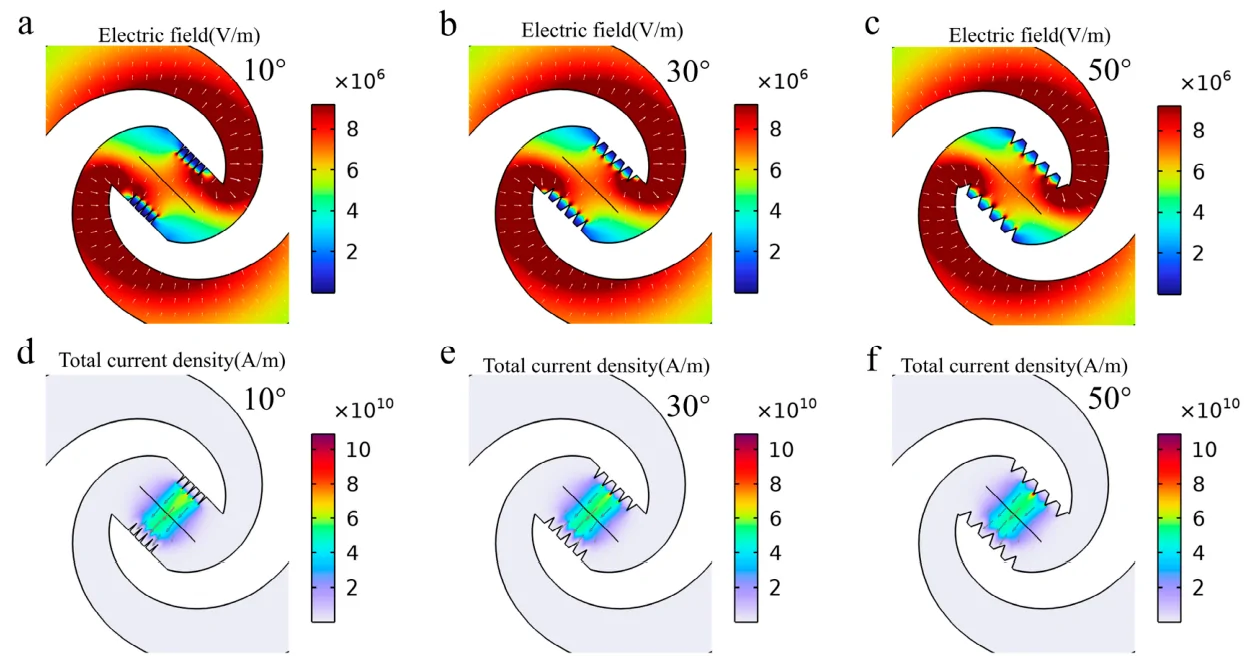
Electric-field and photocurrent-density distributions for selected apex angles: (**a**–**c**) electric field distribution; (**d**–**f**) photocurrent density distribution.

As shown in Figure 3, the electric field strength near the tip increases as the apex angle decreases. This phenomenon is consistent with the electric-field concentration at sharp metallic tips, where surface charges become more concentrated near sharper metal boundaries, resulting in a higher local electric field. However, the apex angle does not only affect the maximum value of the local electric field. Since the tip height is fixed, changing *θ* also alters the lateral width of the tip and the orientation of its two side boundaries. Therefore, the spatial range and orientation of the enhanced electric field near the metal–semiconductor boundary also change accordingly.

For a small apex angle like 10°, although the electric field enhancement is more pronounced, its strong electric field is mainly concentrated in a narrower tip region. In this case, only a portion of the photogenerated carriers can reside within the region of strongest local electric field. As *θ* increases, the lateral dimension of the tip increases, and the lateral extent covered by the two side boundaries within the illuminated area also increases, thereby simultaneously altering the spatial overlap between carriers and the electric field, as well as the distance of nearby photogenerated carriers to the collection boundary. Furthermore, the electric field vector indicates that the direction of the local electric field changes with the direction of the tip boundary. Therefore, the contribution of the tip to the photocurrent depends not only on the magnitude of the local electric field but also on the spatial extent within which the electric field can act on photogenerated carriers and whether it can effectively drive carriers towards the electrode.

**Figure 4:**
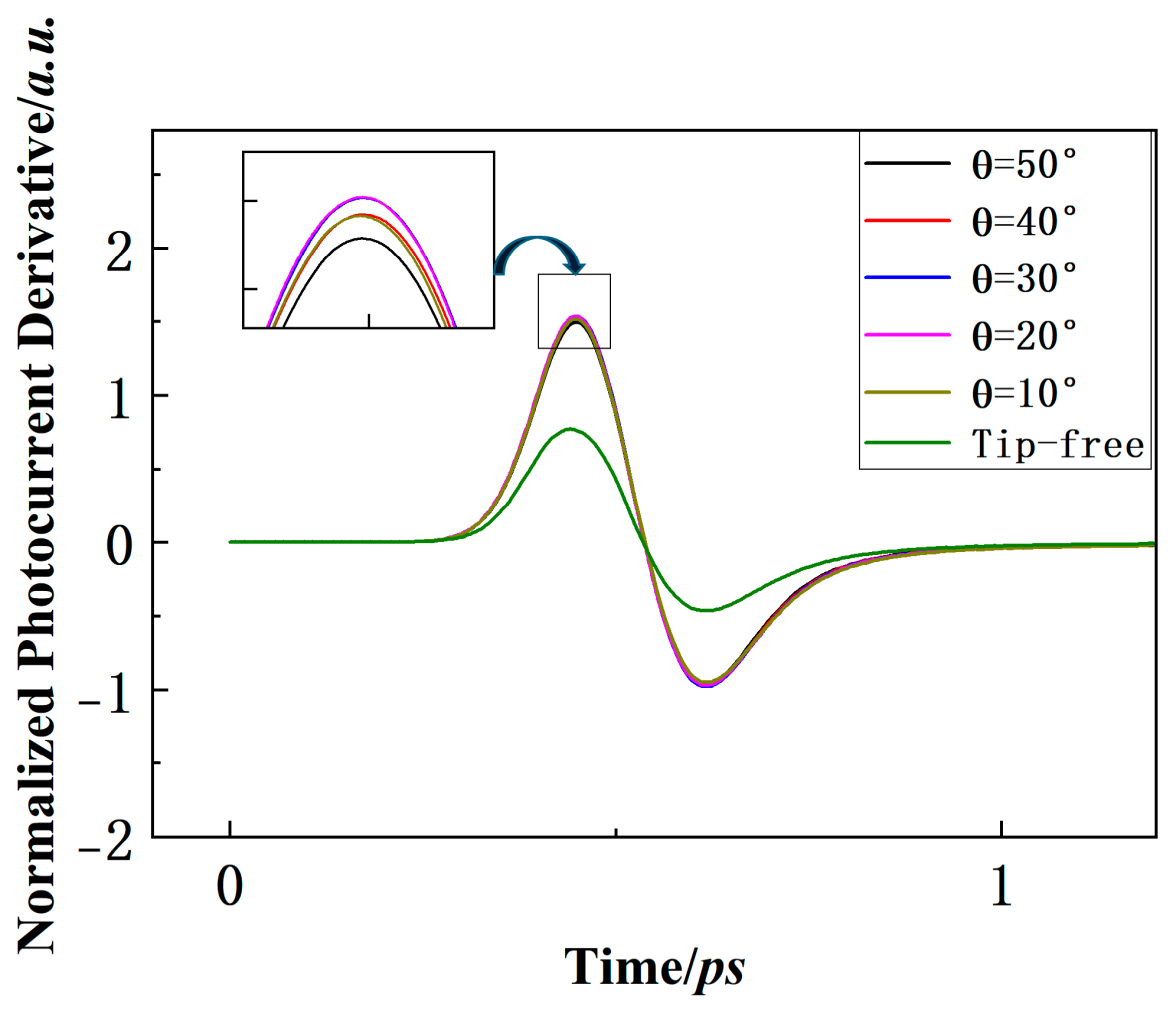
Photocurrent-derivative curves for different tip apex angles and the tip-free reference.

It can be seen from Figure 4 that all tip-array structures exhibit substantially higher photocurrent-derivative responses than the tip-free reference. For *θ* = 10°, 20°, 30°, 40°, and 50°, the positive-peak enhancements are 97.6%, 100.2%, 100.1%, 97.9%, and 94.7%, respectively, while the corresponding peak-to-peak enhancements are 99.9%, 103.2%, 103.9%, 102.4%, and 99.3%. Figure 4 can be understood as the result of the mutual constraints between geometric and electric field factors. At 10°, a strong electric field concentration is accompanied by a narrower effective strong field region. For 20–30°, the tip still has sufficient sharpness to maintain a strong local electric field enhancement, while the wider electrode boundary can form a more balanced spatial interaction with the photogenerated carrier distribution. When the apex angle further increases to 40–50°, the local field concentration capability decreases more significantly, and the expansion of the effective range brought about by the increase in the lateral size of the tip is insufficient to compensate for this reduction in field concentration.

Therefore, the subsequent height and spacing analysis chose *θ* = 30°. Although the responses of 20° and 30° are very close, it is more favorable for the manufacturing tolerance of tip rounding and edge roughness in actual processing.

### 3.3. Influence of Tip Height on the Source-Side Transient Response

We next varied the tip height from *h* = 0.4 to 1.6 μm while fixing *θ* = 30° and *d* = 0.5 μm. Unlike the apex angle, *h* directly controls how far the metal boundary extends into the illuminated gap.

**Figure 5:**
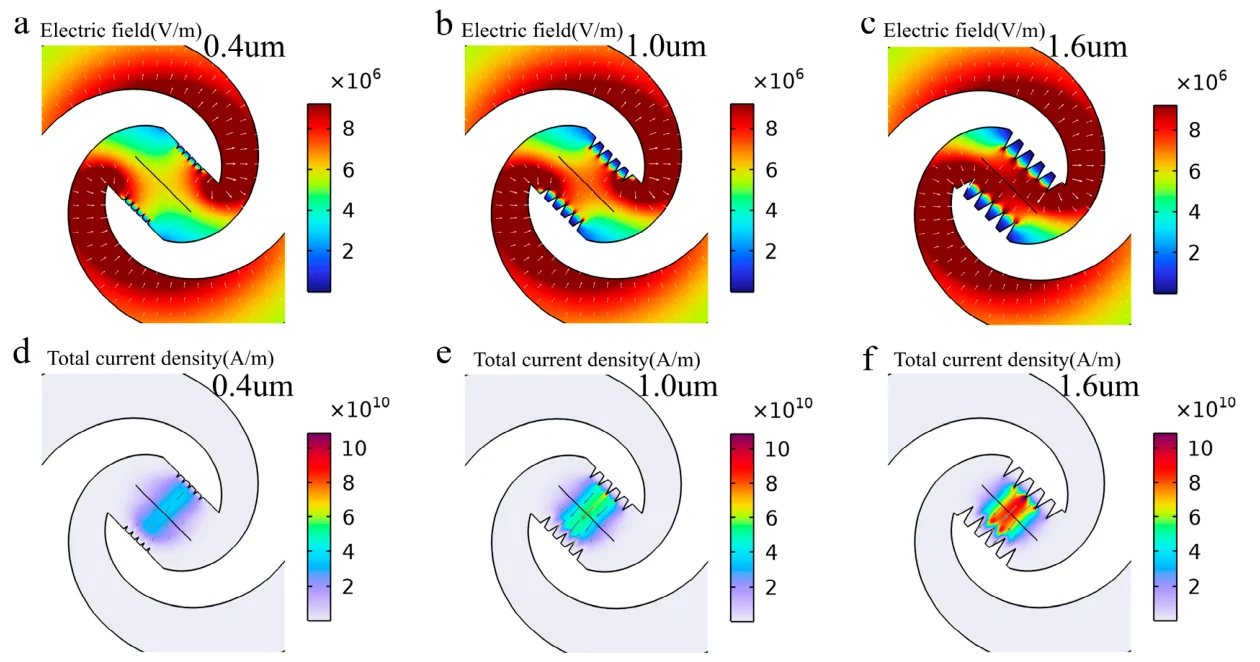
Electric-field and photocurrent-density distributions for selected tip heights: (**a**–**c**) electric field distribution; (**d**–**f**) photocurrent density distribution.

Figure 5 shows that increasing the tip height has a more significant impact on the central active region compared to changing the apex angle. As the tip extends further into the feed gap, the local distance between the metal boundaries decreases. At the same bias voltage, this geometric change enhances the concentration of the local bias electric field and shifts the enhanced electric field region further towards the illumination center. Therefore, more photogenerated carriers can be generated within or closer to the strong bias electric field region.

**Figure 6:**
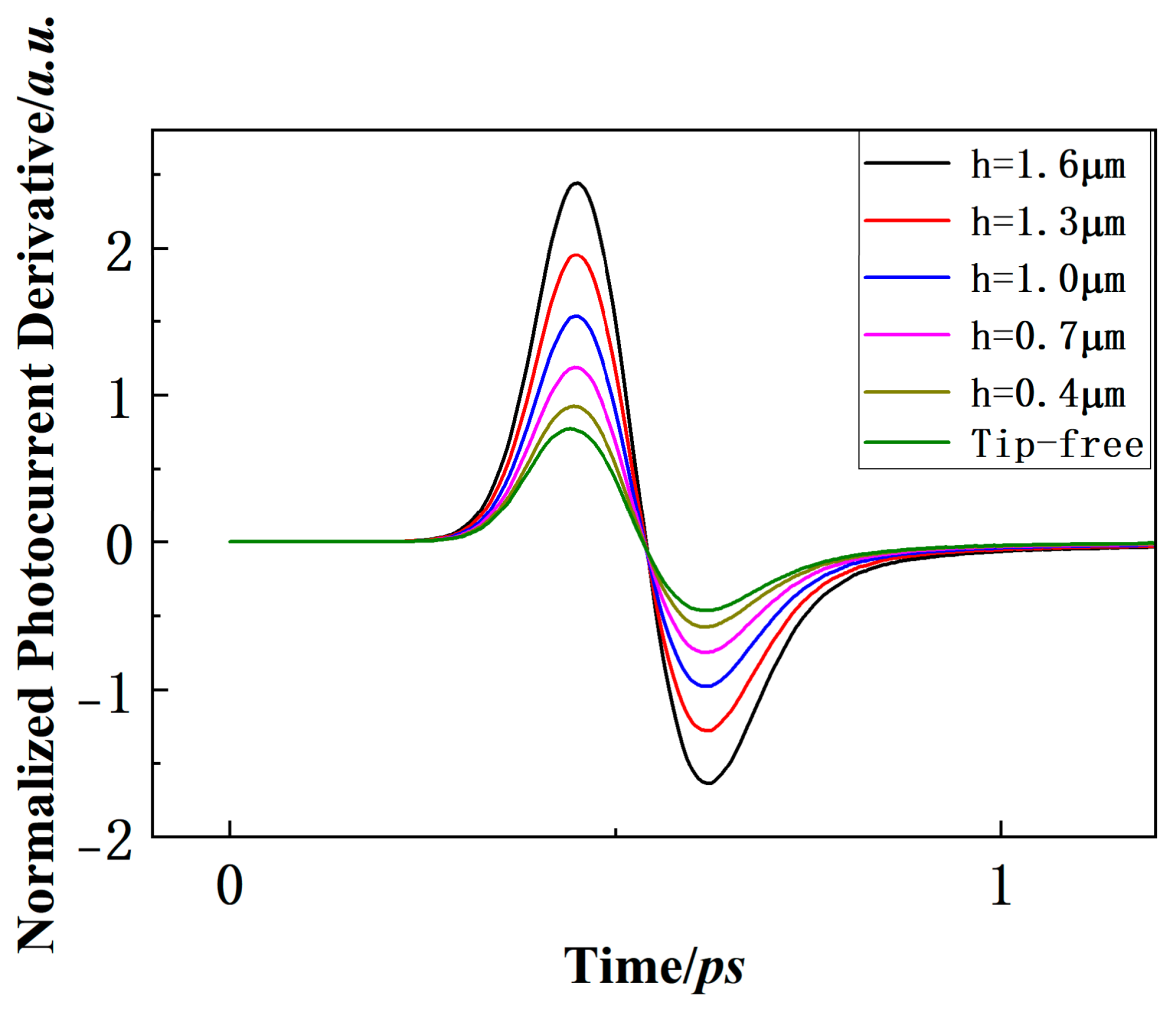
Photocurrent-derivative curves for different tip heights and the tip-free reference.

Furthermore, increasing the height does not merely shift the tip angle forward. While keeping the tip angle constant, the two sides of the tip also lengthen with increasing height, allowing a larger area of the metal–semiconductor collection boundary to extend into the high carrier concentration region. This change simultaneously shortens the effective carrier transport distance and improves the spatial overlap between the collection boundary, the strong bias electric field, and the photogenerated carriers. Therefore, the more pronounced and continuous photocurrent density channel in Figure 6 results from the combined influence of local electric-field enhancement, a reduced local gap, extension of the effective boundary into the active region, and a shortened carrier transport distance.

Figure 6 shows a monotonic increase across the tested range. For *h* = 0.4, 0.7, 1.0, 1.3, and 1.6 μm, the positive-peak enhancements are 19.8%, 54.8%, 100.1%, 154.7%, and 218.8%, and the peak-to-peak enhancements are 21.2%, 57.2%, 103.9%, 162.0%, and 230.9%, respectively. Within the parameter range studied here, the response change caused by height is significantly greater than that caused by angle. This is because changing the tip height directly readjusts the spatial relationship between photogenerated carriers, the high-field region, and the collection boundary, while the change in apex angle mainly affects the sharpness of the tip and the field distribution characteristics locally.

The 1.6 μm design gives the largest source-side response, but it also increases metal coverage and may perturb the feed-region capacitance and antenna impedance. Because the present model does not quantify these electromagnetic characteristics, we regard *h* = 1.6 μm as an upper-response case within the investigated range rather than as an overall optimum. We retained *h* = 1.0 μm for the spacing analysis because it approximately doubles the response with a more moderate geometric perturbation.

### 3.4. Influence of Tip Spacing on the Source-Side Transient Response

Finally, with *θ* = 30° and *h* = 1.0 μm fixed, we varied the tip spacing *d* to 0.25, 0.5, 0.75, and 1.0 μm. It is worth noting here that each electrode has a fixed five tips.

**Figure 7:**
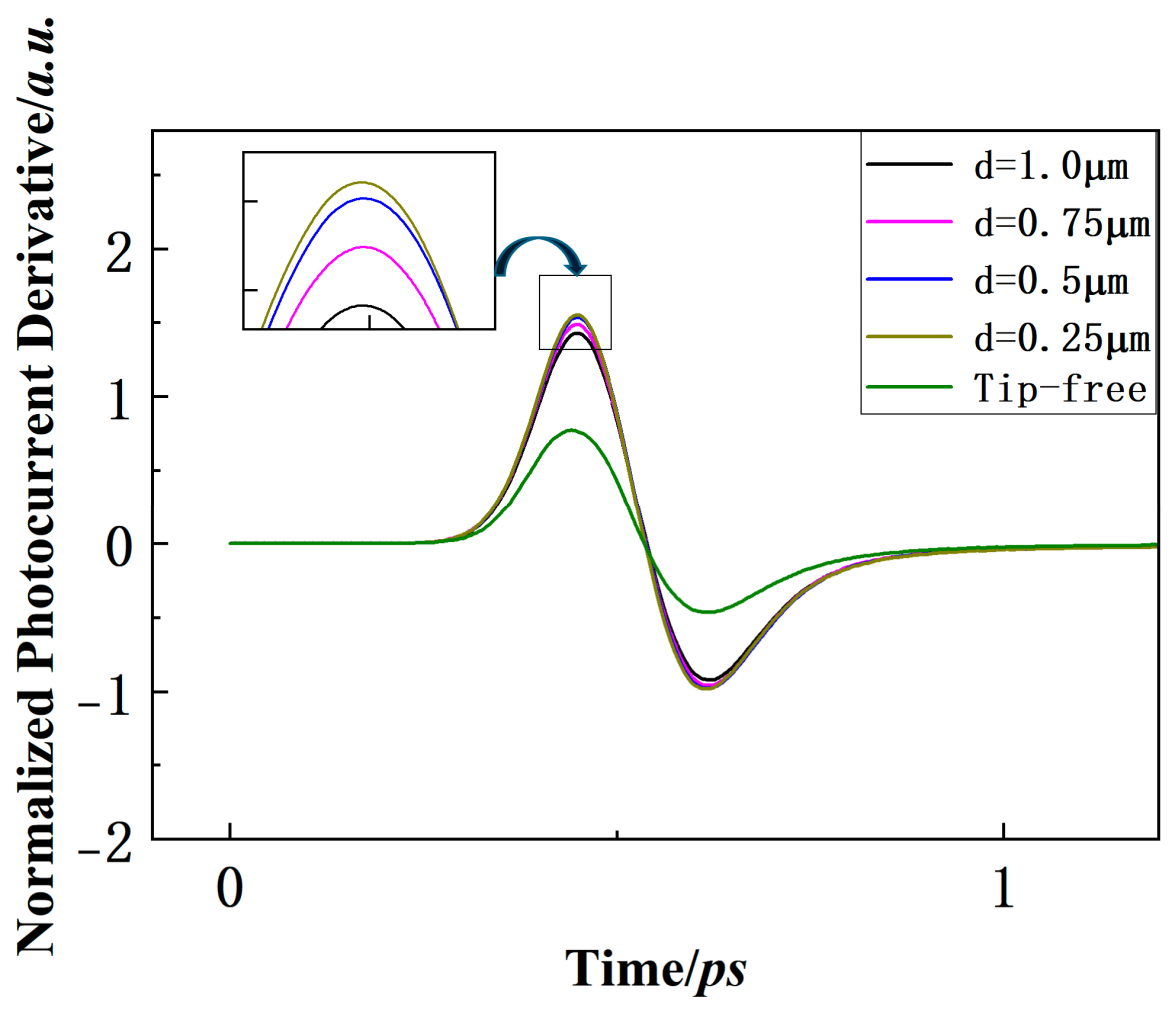
Photocurrent-derivative curves for different tip spacings and the tip-free reference.

Figure 7 shows that the response increases as *d* decreases from 1.0 to 0.5 μm, but changes little between 0.5 and 0.25 μm. For *d* = 0.25, 0.5, 0.75, and 1.0 μm, the positive-peak enhancements are 102.2%, 100.1%, 93.6%, and 85.9%, and the peak-to-peak enhancements are 105.5%, 103.9%, 98.2%, and 90.3%, respectively.

Since the number of tips on each electrode remains fixed at five, changing the spacing not only alters the distance between adjacent tips but also changes the spatial extent of the entire tip array along the feed boundary. Under Gaussian light excitation, the photogenerated carrier concentration is highest near the illumination center and gradually decreases with distance from the center. Therefore, decreasing the spacing causes a larger proportion of the five tips to concentrate in the central high-carrier-concentration region, while increasing the spacing causes the outer tips to gradually move to the region with lower photogenerated carrier concentration.

Therefore, when *d* decreases from 1.0 to 0.5 μm, the improvement in response can mainly be attributed to the improved spatial matching between the tip-induced high-field region and the distribution of photogenerated carriers. When *d* is further reduced from 0.5 to 0.25 μm, the further improvement in response becomes smaller. This indicates that once the central active region is effectively covered by a denser array of tips, further reducing the spacing results in a significant decrease in marginal benefit. In this case, further array compression primarily redistributes the already closely spaced enhanced electric field regions, rather than enabling more newly photogenerated carriers to significantly participate in the effective action of the tip-modulated electric field.

Although *d* = 0.25 μm gives the highest numerical peak, its gain over 0.5 μm is marginal, while the smaller spacing requires tighter lithographic control. We therefore selected *d* = 0.5 μm for the source-enhanced configuration; *d* = 0.75 μm remains a practical alternative when lithographic tolerance has higher priority.

Overall, the source-side enhancement produced by the tip array cannot be simply understood as the result of an increase in the maximum electric field of a single tip. During the drift-diffusion process, the drift current component depends simultaneously on the carrier concentration, carrier mobility, and local electric field vector. Therefore, the transient current is actually determined by the spatiotemporal coupling among the photogenerated carrier distribution, the magnitude and direction of the bias electric field, and the position of the collection boundaries. The three geometric parameters regulate these factors in different ways: *θ* mainly changes the local sharpness of the tip, the lateral geometric size, and the electric field distribution; *h* directly changes the local gap, allowing a larger range of collection boundaries to enter the illuminated active region; *d* changes the spatial position of a fixed number of tips relative to the Gaussian carrier distribution. These factors together determine the evolution of the photocurrent over time and are ultimately reflected in the photocurrent derivative response.

From a practical manufacturing perspective, the micrometer-scale tip array proposed in this paper can be realized through conventional photolithography and metal patterning processes, but several practical manufacturing issues still need to be considered. First, due to limitations in photolithography resolution and metal deposition processes, the actual tip apex may be rounded, especially for structures with smaller apex corners. Simultaneously, edge roughness may alter the local boundary morphology, thereby weakening the local electric field concentration predicted in the ideal sharp tip model. Second, as the tip spacing decreases, the requirements for patterning accuracy further increase, and dimensional deviations are more likely to occur between adjacent tips. Furthermore, tip height, metal thickness, and alignment errors in the central feeding region can all lead to differences between the actual fabricated structure and the ideal numerical model. Therefore, parameter selection not only needs to consider response enhancement but also requires a certain tolerance for manufacturing errors. This is one of the reasons why this paper ultimately adopts *θ* = 30°, *h* = 1.0 μm, and *d* = 0.5 μm as representative design parameters.

### 3.5. Discussions

Local electrode-geometry engineering has been widely investigated as an effective approach for improving the performance of terahertz photoconductive antennas [9,10,11,12,13,14,15,16,17,18]. Previous studies have mainly focused on enhancing optical absorption, increasing local electric-field intensity, or modifying electrode configurations to improve carrier generation and collection. However, most reported structures modify the overall electrode morphology or introduce additional nanoscale optical structures, while the influence of the central feed boundary geometry on the source-side transient response has received relatively limited attention.

Compared to previous methods, our proposed tip array structure introduces only local modifications at the central feed boundary while maintaining the original logarithmic double helix structure. This design strategy minimizes perturbations to the overall antenna geometry and directly optimizes for the regions of photocarrier generation and transient current initial formation.

Table 1 summarizes representative studies related to local structural optimization of photoconductive antennas. These works demonstrate that modifying electrode geometry can effectively improve PCA performance. However, different studies employ different evaluation metrics, including THz electric-field amplitude, generated current, or photocurrent-related parameters; therefore, the enhancement values should be interpreted as a comparison of optimization effectiveness rather than a strict quantitative ranking.

Compared to previous studies, our enhancements are comparable to or better than several reported local structure optimization methods. For the optimized configurations with *θ* = 30°, *h* = 1.0 μm, and *d* = 0.5 μm, the positive peak value and peak-to-peak value of *dI*/*dt* are improved by 100.1% and 103.9%, respectively, compared to the tipless reference structure.

More importantly, the main contribution of this work is not only the response enhancement itself, but also the systematic investigation of how different geometric parameters influence the source-side response. As demonstrated in Section 3.2, Section 3.3 and Section 3.4, the apex angle mainly affects the local field concentration, whereas the tip height dominates the response variation by simultaneously modifying the effective interaction region and the relative position between the electric-field distribution and photogenerated carriers. The spacing parameter further determines the spatial coverage of the tip array, with excessive spacing reducing the contribution of tips located away from the active carrier-generation region.

Several experimental studies have demonstrated that local electrode engineering, such as plasmonic electrodes and microstructured contacts, can improve the output performance of photoconductive antennas [12,13,14,15,16,17,18], supporting the feasibility of boundary-geometry optimization strategies. Although the present work focuses on the source-side transient response based on a semiconductor model and does not include full electromagnetic radiation analysis or experimental verification, the obtained results provide a theoretical basis for future fabrication and experimental evaluation of the proposed structure.

Experimental studies on plasmonic and microstructured photoconductive antennas have demonstrated that local electrode engineering can effectively improve THz emission performance. For example, plasmonic contact electrodes and nano-engineered electrode structures have achieved significant enhancement in practical devices by increasing optical absorption and modifying carrier transport paths. Although the present work focuses on source-side transient response based on semiconductor transport simulation rather than direct experimental radiation measurement, these experimental results support the feasibility of local electrode optimization and provide a basis for future fabrication and verification of the proposed tip-array structure.

## 4. Conclusions

We investigated the impact of a micrometer-scale tip array with a central feed boundary introduced into a logarithmic double-helix photoconductive antenna on the transient response at the source end based on a transient semiconductor model. Through the photocurrent derivative response, we systematically analyzed the influences of tip angle, height, and spacing on the antenna’s transient response.

The results show that the tip array can effectively modulate the local electric field distribution and improve the spatial interaction between the bias electric field and photogenerated carriers. The optimized structure achieves a 103.9% peak-to-peak enhancement of the photocurrent derivative compared to the tipless reference structure. Among the parameters, the tip height has the most significant impact on the transient response, while the tip angle and spacing mainly affect the local electric field distribution and the spatial range of the tip array.

Unlike previous studies that primarily focused on the enhancement influences brought about by specific electrode structures, this paper systematically analyzes the relationship between the geometric parameters of the tip array and the transient response of the logarithmic double-helix photoconductive antenna source, further revealing the role of local electrode boundary morphology in modulating transient carrier dynamics.

Future work will focus on the experimental fabrication and testing of optimized tip array structures to further validate the predicted source-side transient response enhancement. Simultaneously, full-wave electromagnetic simulations will be used to investigate the impact of tip array parameters on impedance matching, frequency response, and far-field THz radiation characteristics. Furthermore, the influence of different tip distributions and geometric parameters on the performance of the photoconductive antenna will be further studied to gain a deeper understanding of the relationship between electrode boundary design and device performance.

## Figures and Tables

**Table 1:** Comparison of representative structural optimization strategies for terahertz photoconductive antennas.

Structural Modification	Main Optimization Target	Evaluation Metric	Enhancement	Reference
Plasmonic contact electrodes	Optical absorption enhancement	THz emission intensity	Significant enhancement	[12]
Sawtooth electrode edges	Local electric-field enhancement	Peak THz electric field	~40%	[18]
Buried metallic rod structures	Carrier generation and collection	THz current	~82%	[26]
Central-feed-boundary tip array	Source-side transient response	Peak-to-peak d*I*/d*t*	103.9%	This work

## Data Availability

The raw data supporting the conclusions of this article will be made available by the authors on request.
